# 

**Published:** 1836-10-01

**Authors:** 


### CONTENTS

OF THE

### MEDICO-CHIRURGICAL REVIEW,

### No. L. OCTOBER 1, 1836,

### [BEING No. X. OF A DECENNIAL SERIES.]

### REVIEWS.

I.

PATHOLOGY OF THE NERVOUS SYSTEM.

1. Lectures on the Nervous System. By MARSHALL HALL, M.D. F.R.S. &c. .. } 293
2. Outlines of Human Pathology. By HERBERT MAYO, F.R.S. .. .. .. .. .. }

A. Pathology of the Nervous System .. .. .. .. .. .. .. .. .. .. 295
B. Palsy .. .. .. .. .. .. .. .. .. .. .. .. .. .. .. .. 295
C. Apoplexy .. .. .. .. .. .. .. .. .. .. .. .. .. .. .. 307
D. Accidental Apoplectic Symptoms .. .. .. .. .. .. .. .. .. .. 310
E. Concussion and Compression .. .. .. .. .. .. .. .. .. .. .. 312
F. Epilepsy .. .. .. .. .. .. .. .. .. .. .. .. .. .. .. 315

II.

Stammering considered with reference to its Cure. By RICHARD CULL .. .. .. 319

III.

The Proofs of Infanticide considered. By WILLIAM CUMMIN, M.D. .. .. .. .. 322
The Hydrostatic Test .. .. .. .. .. .. .. .. .. .. .. .. .. 323

IV.

The Report of a Committee on the New Poor Law Act, appointed by the Provincial Medical and Surgical Association, at the Anniversary Meeting held at Oxford, and read at the Anniversary Meeting, held at Manchester, July 21, 1836 .. .. .. 325

V.

De la Prostitution dans la Ville de Paris, &c. By M. PARENT DUCHATELET, M.D. .. 333
A. Manners and Habits of Prostitutes .. .. .. .. .. .. .. .. .. .. 335
B. Physiological Considerations .. .. .. .. .. .. .. .. .. .. .. 339

VI.

Observations on Rheumatism, and on Burning of the Feet. By Mr. MALCOLMSON .. 341

VII.

On the remedial Agency of the Air Pump. By Sir JAMES MURRAY, M.D. .. .. .. 346

VIII.

The Transactions of the Provincial Association. [Concluding Notice.] .. .. .. 347
A. Mr. Chavasse on the Injurious Effects of the Secale Cornutum .. .. .. 349
B. Extensive Disease of the Female Pudendum. By T. Brayne, Esq.. .. .. 352
C. Case of Neuralgia of the Uterus during Pregnancy. By J. Holbrook, M.D.. 354
D. Mr. Coley on Hydrometra.. .. .. .. .. .. .. .. .. .. .. .. 357
E. Records of Ovarian Tumor. By Dr. Barlow .. .. .. .. .. .. .. .. 360
F. Case of Fungoid Tumor, succeeded by Melanosis. By Dr. Norris .. .. 368

IX.

A Practical Treatise on Urethritis and Syphilis. By WM. HENRY JUDD. [Concluding Notice] .. .. .. .. .. .. .. .. .. .. .. .. .. .. .. .. .. .. 370

Venereal Eruptions .. .. .. .. .. .. .. .. .. .. .. .. .. .. .. .. 373

A. Venereal Exanthemata .. .. .. .. .. .. .. .. .. .. .. .. 375

B. Papulæ .. .. .. .. .. .. .. .. .. .. .. .. .. .. .. 377

C. Vesiculæ Venereæ .. .. .. .. .. .. .. .. .. .. .. .. .. 383

D. Rupia Venerea .. .. .. .. .. .. .. .. .. .. .. .. .. 387

E. Venereal Pustules .. .. .. .. .. .. .. .. .. .. .. .. .. 391

F. Maculæ Venereæ .. .. .. .. .. .. .. .. .. .. .. .. .. 398

G. Venereal Tubercles .. .. .. .. .. .. .. .. .. .. .. .. 399

H. Squamæ Venereæ .. .. .. .. .. .. .. .. .. .. .. .. .. 401

X.

Homœopathy and Allopathy? or large, small, and atomic Doses. By D. UWINS, M.D. 405

XI.

The Anatomist's Instructor, and Museum Companion. By FRED. JOHN KNOX .. .. 408

XII.

Five Cases of Ununited Fracture, in which Excision of the Ends of the Bone, or the Seton was employed .. .. .. .. .. .. .. .. .. .. .. .. .. .. .. 428

### PERISCOPE.

### PART I.

### Spirit of the English Periodicals, and Notices of English Medical Literature.

1. On Antimony in Delirium of Fevers. By R. Graves, M.D. .. .. .. .. .. .. 437
2. Gun-shot Wounds of the Face and Neck .. .. .. .. .. .. .. .. .. .. .. .. 441
3. Pathology puzzled .. .. .. .. .. .. .. .. .. .. .. .. .. .. .. .. .. .. 443
4. Enuresis .. .. .. .. .. .. .. .. .. .. .. .. .. .. .. .. .. .. .. .. 444
5. Alexander Lee's Celsus, with Notes .. .. .. .. .. .. .. .. .. .. .. .. .. 446
6. Contributions to Pathology. By W. Smith .. .. .. .. .. .. .. .. .. .. .. 446
7. The Skull of Spurzheim .. .. .. .. .. .. .. .. .. .. .. .. .. .. .. .. 448
8. Dr. Middlemore on Pterygium .. .. .. .. .. .. .. .. .. .. .. .. .. .. 449
9. Chronic Laryngitis. By Dr. Roots .. .. .. .. .. .. .. .. .. .. .. .. .. 449
10. Fatal Case of Chorea .. .. .. .. .. .. .. .. .. .. .. .. .. .. .. .. 451
11. Extraction of a Pin from the Female Bladder by a new Instrument .. .. .. 452
12. Temporary Loss of Memory after an Injury of the Head .. .. .. .. .. .. .. 453
13. Acute Pericarditis. By Dr. Roots .. .. .. .. .. .. .. .. .. .. .. .. .. 455
14. The Statistics of Hernia .. .. .. .. .. .. .. .. .. .. .. .. .. .. .. 457
15. Tight Lacing .. .. .. .. .. .. .. .. .. .. .. .. .. .. .. .. .. .. .. 460
16. Poisoning by Cantharides .. .. .. .. .. .. .. .. .. .. .. .. .. .. .. 461
17. M. Breschet's Operation for Varicocele .. .. .. .. .. .. .. .. .. .. .. 463
18. Irish Unanimity; or the Blessings of Medical Corporations .. .. .. .. .. .. 465
19. Lithotomy and Lithotrity .. .. .. .. .. .. .. .. .. .. .. .. .. .. .. 465
20. Case of Dropsy with Ovarian Tumor, by William Coulson; with an Analysis of the Fluid, by Dr. Bostock .. .. .. .. .. .. .. .. .. .. .. .. .. .. 473
21. On Mercury in Onychia Maligna. By Dr. Beck .. .. .. .. .. .. .. .. .. 474
22. Lithotomy à deux temps, on a Child .. .. .. .. .. .. .. .. .. .. .. .. 476

### Bibliographical Notices.

23. On Deformities of the Chest. By Mr. Coulson .. .. .. .. .. .. .. .. 477
24. An Inquiry into Puerperal Fever. By Mr. Moore .. .. .. .. .. .. .. .. 478
25. On the Antidotal Treatment of the Epidemic Cholera. By John Parkin .. .. 480
26. The Practical Anatomy of the Nervous System. By F. Le Gros Clark.. .. .. 481

PART II.

### Spirit of the Foreign Periodicals, &c.

1. On the Varieties of the Human Race.. .. .. .. .. .. .. .. .. .. .. .. 485
2. M. Serres on progressive Development in the Higher Animals .. .. .. .. 488
3. The Siamese Twins .. .. .. .. .. .. .. .. .. .. .. .. .. .. .. .. 493
4. Case of Twins; the second Child born 4 days after the first .. .. .. .. .. 495
5. Remarks on Osteo-Malacia, with Cases .. .. .. .. .. .. .. .. .. .. .. .. 496
6. Radical Cure of Hernia .. .. .. .. .. .. .. .. .. .. .. .. .. .. .. 500
7. The Small-pox, as observed at Vienna in 1835 .. .. .. .. .. .. .. .. .. 500
8. Case of Sternutation cured by a Pinch of Snuff.. .. .. .. .. .. .. .. .. 501
9. Remarks on a certain Form of partial Convulsion .. .. .. .. .. .. .. .. 501
10. Severe Symptoms from the Bite of a Spider .. .. .. .. .. .. .. .. .. .. 508
11. Poisoning from a Viper-bite—Cure with Sulphate of Quinine.. .. .. .. .. .. 509
12. Remarks on the frequency of Gangrene of the Lungs in the Insane .. .. .. 509
13. Injections of Gaseous Chlorine in Hydrocele—Treatment of Chilblains .. .. .. 513
14. Cure of an obstinate Ozœna .. .. .. .. .. .. .. .. .. .. .. .. .. .. 514
15. On the Coincidence of Endocarditis and of Pericarditis with Acute Rheumatism .. 514
16. Cases of Glanders in the Human Subject .. .. .. .. .. .. .. .. .. .. 516
17. Hydrated Peroxide of Iron, an Antidote to Arsenic .. .. .. .. .. .. .. .. 518
18. Congenital Occlusion of the Vagina cured by a new Method .. .. .. .. .. .. 522
19. Singular cases of Congenitally Imperforate Vagina—Menstruation by the Urethra—Successful Operation .. .. .. .. .. .. .. .. .. .. .. .. .. .. .. 526
20. Human Embryo vomited by a Child .. .. .. .. .. .. .. .. .. .. .. .. 527
21. On the more common Causes of Death in New-born Infants .. .. .. .. .. 527
22. Treatment of Ossification occurring in Muscular Texture .. .. .. .. .. .. 527

PART III.

### Clinical Review, and Hospital Reports.

Birmingham Infirmary.

1. Report of the Out-cases attended by George Parsons, Esq. in 1834-5 .. .. } 528
2. Report of the Out-patients attended by Mr. Ryland, in 1834-5 .. .. .. }

St. Thomas's Hospital.

3. St. Thomas's Hospital Reports by J. F. South .. .. .. .. .. .. .. .. .. 534
4. Phlebitis .. .. .. .. .. .. .. .. .. .. .. .. .. .. .. .. .. .. .. 535
5. Stone in the Bladder .. .. .. .. .. .. .. .. .. .. .. .. .. .. .. .. 537
6. Medullary Carcinoma .. .. .. .. .. .. .. .. .. .. .. .. .. .. .. .. 544
7. Mortification of Hernia .. .. .. .. .. .. .. .. .. .. .. .. .. .. .. 546

Belfast Fever Hospital.

8. Statistics of Fever .. .. .. .. .. .. .. .. .. .. .. .. .. .. .. .. .. 547

London Hospital.

9. Statistics of the London Hospital, by T. Edmonds, Esq. .. .. .. .. .. .. 558

PENNSYLVANIA HOSPITAL:

10. Reports of Surgical Cases, by T. Kirkbride, M.D. .. .. .. .. .. .. .. .. 560

BLOCKLEY HOSPITAL.

11. Dr. Gerhard's Reports on Pneumothorax .. .. .. .. .. .. .. .. .. .. 562

GUY'S HOSPITAL.

12. Analysis of the Organic Lesions found in 100 Cases, in which the Urine was Albuminous .. .. .. .. .. .. .. .. .. .. .. .. .. .. .. 564
   - A. Lesions in the Serous Membranes .. .. .. .. .. .. .. .. .. 565
   - B. Lesions of the Heart .. .. .. .. .. .. .. .. .. .. .. .. 565
   - C. Lesions of the Lungs .. .. .. .. .. .. .. .. .. .. .. .. 566
   - D. Lesions of the Liver .. .. .. .. .. .. .. .. .. .. .. .. 566
   - E. Lesions of other Abdominal Viscera .. .. .. .. .. .. .. .. 567
   - F. Lesions of the Brain .. .. .. .. .. .. .. .. .. .. .. .. 567
   - G. Immediate Causes of Death .. .. .. .. .. .. .. .. .. .. .. 567
   - H. Age at which Death occurs .. .. .. .. .. .. .. .. .. .. .. 567

### Miscellanies.

1. An Act to remunerate Medical Witnesses at Coroner's Inquests .. .. .. .. 568
2. Provincial Medical and Surgical Association .. .. .. .. .. .. .. .. 570
3. New Farthing Club; or Metropolitan Infinitesimal Dispensary.. .. .. .. .. 571
4. Hæmorrhage from the Medulla of the Humerus after Amputation .. .. .. .. 573
5. Sixteenth Report of the Dundee Lunatic Asylum—Effect of Fright.. .. .. .. 574
6. Medical Clubs .. .. .. .. .. .. .. .. .. .. .. .. .. .. .. .. 574
7. Patronage of Quacks .. .. .. .. .. .. .. .. .. .. .. .. .. .. 576
8. Spontaneous Combustion .. .. .. .. .. .. .. .. .. .. .. .. .. 577
9. Tunnels on Railroads .. .. .. .. .. .. .. .. .. .. .. .. .. .. 577

BIBLIOGRAPHICAL RECORD .. .. .. .. .. .. .. .. .. .. .. .. .. .. 579

### Extra-Limites.

1. Dr. Ryan's Formulary of Hospitals .. .. .. .. .. .. .. .. .. .. .. 581
2. Charter of the New London University .. .. .. .. .. .. .. .. .. .. 583
3. Unfermented Bread.. .. .. .. .. .. .. .. .. .. .. .. .. .. .. .. 586
4. Dr. Bright on Diseased Kidnies—Mr. Barlow's Letter respecting .. .. .. .. 586
5. Gold-pointed Lancet .. .. .. .. .. .. .. .. .. .. .. .. .. .. .. 587
6. New and Nutritious Aliment—Tous-les-Mois .. .. .. .. .. .. .. .. 587
7. Extracts from a Petition to Parliament of the Medical Professors of the Andersonian University, for an Extension of University Reform .. .. .. .. .. .. .. 588

